# Histopathological characterization of corrosion product associated adverse local tissue reaction in hip implants: a study of 285 cases

**DOI:** 10.1186/s12907-016-0025-9

**Published:** 2016-02-27

**Authors:** Benjamin F. Ricciardi, Allina A. Nocon, Seth A. Jerabek, Gabrielle Wilner, Elianna Kaplowitz, Steven R. Goldring, P. Edward Purdue, Giorgio Perino

**Affiliations:** Department of Orthopedic Surgery, Hospital for Special Surgery, New York, NY USA; Healthcare Research Institute, Hospital for Special Surgery, New York, NY USA; Division of Research, Hospital for Special Surgery, New York, NY USA; Department of Pathology and Laboratory Medicine, Hospital for Special Surgery, 535 East 70th Street, New York, NY 10021 USA

**Keywords:** Adverse local tissue reaction, Corrosion products, Revision arthroplasty, Synovial inflammation, Metal-on-metal total hip replacement, Hip resurfacing

## Abstract

**Background:**

Adverse local tissue reaction (ALTR), characterized by a heterogeneous cellular inflammatory infiltrate and the presence of corrosion products in the periprosthetic soft tissues, has been recognized as a mechanism of failure in total hip replacement (THA). Different histological subtypes may have unique needs for longitudinal clinical follow-up and complication rates after revision arthroplasty. The purpose of this study was to describe the histological patterns observed in the periprosthetic tissue of failed THA in three different implant classes due to ALTR and their association with clinical features of implant failure.

**Methods:**

Consecutive patients presenting with ALTR from three major hip implant classes (*N* = 285 cases) were identified from our prospective Osteolysis Tissue Database and Repository. Clinical characteristics including age, sex, BMI, length of implantation, and serum metal ion levels were recorded. Retrieved synovial tissue morphology was graded using light microscopy. Clinical characteristics and features of synovial tissue analysis were compared between the three implant classes. Histological patterns of ALTR identified from our observations and the literature were used to classify each case. The association between implant class and histological patterns was compared.

**Results:**

Our histological analysis demonstrates that ALTR encompasses three main histological patterns: 1) macrophage predominant, 2) mixed lymphocytic and macrophagic with or without features of associated with hypersensitivity/allergy or response to particle toxicity (eosinophils/mast cells and/or lymphocytic germinal centers), and 3) predominant sarcoid-like granulomas. Implant classification was associated with histological pattern of failure, and the macrophagic predominant pattern was more common in implants with metal-on-metal bearing surfaces (MoM HRA and MoM LHTHA groups). Duration of implantation and composition of periprosthetic cellular infiltrates was significantly different amongst the three implant types examined suggesting that histopathological features of ALTR may explain the variability of clinical implant performance in these cases.

**Conclusions:**

ALTR encompasses a diverse range of histological patterns, which are reflective of both the implant configuration independent of manufacturer and clinical features such as duration of implantation. The macrophagic predominant pattern and its mechanism of implant failure represent an important subgroup of ALTR which could become more prominent with increased length of implantation.

## Background

The introduction over the past two decades of alternative bearing surfaces, in particular a new generation of metal-on-metal (MoM) bearing, and increased modularity at the head-neck and neck-stem tapers has attempted to reduce wear debris formation at the bearing surface, risk of dislocation, and improve accurate reproduction of leg length, offset, and version [1–3]. These modifications have had unintended consequences, although clinical concerns regarding formation of corrosion products were raised; in particular, increased rates of adverse periprosthetic soft tissue reactions reported across a diverse spectrum of implant configurations [4–9]. These failures have resulted in extensive soft tissue necrosis, injury to the hip abductors, increased revision complications, and significant patient morbidity [8, 10–12].

Early studies described an unusual pattern of periprosthetic soft tissue inflammation with mixed macrophagic and lymphocytic infiltrates, variable tissue necrosis, vascular wall changes, and cytoplasmic inclusions of uncertain composition in the macrophages, which was collectively described as aseptic lymphocyte dominated vasculitis associated lesion (ALVAL) [13–15]. The formation of corrosion products at modular junctions and/or bearing surface and subsequent penetration into the periprosthetic soft tissue have been a common feature associated with the reaction [5, 16–18]. More recent studies have focused on characterizing the lymphocytic infiltrate, noting mixed interstitial and perivascular B- and T-cell populations with formation of germinal centers or sarcoid-like granulomas in subsets of patients [17, 19, 20].

Unlike the early reports that focused primarily on aspects of lymphocytic infiltrate and necrosis, subsequent studies suggested that the histological spectrum of these reactions, named adverse local tissue reaction (ALTR) or adverse reaction to implant debris (ARMD) is more diverse than originally appreciated, and lymphocyte rich infiltrate with significant necrosis represents only a subset of these cases [17, 20, 21]. In particular, a subgroup of patients with neo-synovial florid macrophagic infiltrate containing wear debris with no or minimal lymphocytic component in their periprosthetic tissue has been described in these studies, although its contribution to implant failure has not been well characterized. It is critical to identify the full spectrum of ALTR failures because different histological subtypes may have unique needs for longitudinal clinical follow-up and complication rates after revision arthroplasty.

In the present study, we report the histological features of 285 cases of ALTR from a large, diverse group of hip implants that includes three major classes: metal-on-metal (MoM) hip resurfacing arthroplasty (HRA), MoM large head total hip arthroplasty (THA), and non-MoM THA with cobalt/chrome (CoCr) dual modular neck. Histopathological analysis of the periprosthetic tissue across the three classes of implants was performed to answer the following research questions: 1. What are the histopathological patterns of soft tissue failure in ALTR; 2. What is the association between implant class and different histopathological features of ALTR; 3. What is the association of histopathological findings with clinical features of implant failure.

## Methods

### Patients

**Fig. 1:**
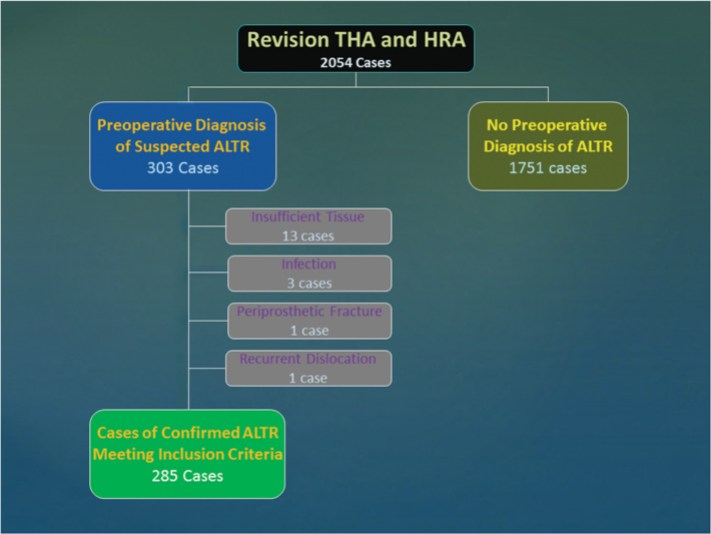
Flowchart of case study selection. The flowchart summarizes the process for inclusion and exclusion criteria for the study during the time period June 2011-December 2014, starting with the total number of THA and HRA revisions performed during the study period and ending with the final number of cases examined

All patients who underwent revision hip arthroplasty between June 2011 and December 2014 implanted with a prosthetic device of the above mentioned classes of implants at risk of ALTR were identified retrospectively from the prospective Osteolysis/Adverse Local Reaction Tissue Database and Repository at our institution (Fig. 1). These patients were all eligible for inclusion in the current study [*N* = 303]. Exclusion criteria included infection diagnosed in compliance with the criteria reported by the International Consensus Meeting on periprosthetic joint infection and accepted by the Centers for Disease Control [22] [*N* = 3] with 5 out of 5, 6 out of 6, and 5 out of 5 intraoperative positive cultures, insufficient tissue retrieval for comparative pathologic examination (less than 5 tissue sections and more than 75 % tissue necrosis at light microscopy examination on all slides examined) [*N* = 13], and two cases for non-ALTR related post-operative complications with histological examination: periprosthetic fracture [*N* = 1] and recurrent dislocation [*N* = 1]. The exclusion of these patients left a total of 285 cases for inclusion in this study. In addition, 18 cases were identified with a post-operative unexpected diagnosis of ALTR in conventional MoP implants without dual modular neck that were not consented for inclusion in our registry prospectively and were not eligible for enrollment in the current study.

Patients were divided into three groups based on the design of their implant. Previous work has shown that implant design influences both clinical and pathologic manifestations of ALTR [17]. The three major implant classes examined were: 1. MoM HRA group; 2. MoM large head (≥36 mm) THA with or without cobalt chromium (CoCr) metallic adapter sleeve (MoM LHTHA group); and 3. Metal-on-polyethylene (MoP), ceramic-on-polyethylene (CoP), or ceramic-on-ceramic (CoC) bearing surface with femoral heads <36 mm and (CoCr) dual modular neck (Non-MoM DMNTHA). These represent the most common implant classes that have resulted in ALTR in case reports and case series [5, 23–27]. Demographic data (age, sex, body mass index, duration of implantation, duration of symptoms, implant type) were recorded for each patient when available. The onset of symptoms was assessed via questionnaire at the time of revision surgery. Symptoms included increasing pain around the hip and mechanical symptoms such as “grinding sensation”. Other symptoms such as discomfort around the hip, although frequent in the Non-MoM DMNTHA group were not considered positive unless progression to pain was recorded before revision. Preoperative serum cobalt and chromium levels were obtained by quantitative inductively coupled plasma-mass spectrometry at the operating surgeons’ discretion (ARUP Laboratories, Salt Lake City, Utah). Ethical committee approval was obtained prior to this study and all patients had an informed consent obtained in writing for inclusion in the registry (Institutional Review Board, Hospital for Special Surgery, Protocol Number 26085).

### Tissue collection and sampling

Tissue collection and sampling for all patients was performed as previously described [17]. Briefly, patients suspected of having ALTR underwent magnetic resonance imaging (MRI) with multi-acquisition variable-resonance image combination (MAVRIC) scan to further reduce susceptibility artifact. Areas of inflammation were identified preoperatively on MRI when available, and used as guidance for tissue sampling by the operating surgeon. Samples were taken from multiple regions around the hip joint including the periprosthetic pseudocapsule, bursal synovium, and adjacent skeletal muscle when necessary and labeled accordingly. Acetabular and femoral bone samples, core biopsies of osteolytic areas, and/or reamings were collected at the discretion of the operating surgeon to evaluate possible bone marrow involvement when suitable. Extensive sampling was performed at macroscopic examination with care to the orientation of the specimens, including necrotic areas and/or friable, loose material. Femoral heads from resurfacing specimens were separated from the metallic cup at surgery when possible and extensively sampled or subject to multiple biopsies when retrieved in situ. The mean number of individual surgical specimens between the groups was not different [DMN cohort was 4.3 (SD 1.5), for the MoM THA cohort was 3.5 (1.6), and for the resurfacing cohort 3.5 (1.3); *p* > 0.05]. Extensive samples between 5 and 15 tissue blocks containing one or two histological sections were taken depending on the available tissue for each specimen.

### Histological analysis

Histological analysis was performed as previously described [17]. Briefly, all sections were processed and embedded with standard procedures, stained routinely with hematoxylin-eosin. Cases were scored for this study by an experienced musculoskeletal pathologist (GP) and a surgeon trained in examining periprosthetic tissue from revision hip arthroplasty (BFR). Investigators were blinded from clinical patient characteristics. All cases were examined by both observers. Disagreement was handled by consensus between the two observers. This method of grading and assessment has been reported in previous publication [17] and also validated for intraobserver variability [28]. The ALVAL scoring system proposed by Campbell et al, which was previously used as correlative index with MRI imaging analysis, was recorded for each case [13, 28].

Histological sections were examined for synovial structure, cellularity, macrophage particle content, and bone marrow involvement using a previously described scoring system [17] and summarized in Table 1. Results were expressed as the percentage of samples containing the selected feature. All patients enrolled in our registry over the same time period with a diagnosis of aseptic loosening due to osteolysis with conventional MoP implants without dual modular neck (MoP OLTHA) were subject to the same tissue collection, histological analysis, and scored to serve as non-ALTR controls for pathological data [*N* = 31].

**Table 1:** Histological grading system used for all cases of ALTR

Synovial Structure	Cellularity	Macrophage Content	Bone and Bone Marrow Involvement
Synovial Layer Loss (Present, Absent)	Macrophages (Grade 0–3)	Polyethylene Particles (Present, Absent)	Necrosis (Present, Absent)
Cell Exfoliation (Present, Absent)	Lymphocytes (Grade 0–4)	Metal Particles (Present, Absent)	Macrophages (Present, Absent)
Soft Tissue Necrosis (Present, Absent)	Stromal Cells (Grade 1–3)	Corrosion Products (None, Intracellular, Extracellular)	Reactive Lymphocytic Aggregates (Present, Absent)
Vascular Wall Changes (Present, Absent)	Neutrophils (Present, Absent)		Germinal Centers (Present, Absent)
Granulomas (Present, Absent)	Plasma Cells (Grade 0–2)		
	Eosinophils (Present, Absent)		

### Histological patterns

Several histological patterns have been observed in ALTR in previous studies [13, 17, 20, 21, 29, 30]. We divided these into four broad groups based on these previous studies: 1) predominantly macrophagic pattern with absent or minimal lymphocytic response, 2) mixed inflammatory pattern, macrophagic and lymphocytic with variable presence of plasma cells, eosinophils, and mast cells, and 3) granulomatous pattern, predominant or associated with the mixed inflammatory pattern; and 4) predominantly lymphocytic pattern with absence of macrophagic component [Table 2]. The macrophagic pattern represents a group of patients with an adverse soft tissue reaction resulting in implant failure with minimal lymphocytic infiltration [20, 30, 31]. The mixed inflammatory group is divided into two subsets: (A) with and (B) without lymphocytic germinal centers usually associated with tall endothelial cell venules and/or mast cell/eosinophilic infiltrate because the A subset may identify patients with distinct immunologic response [13, 15, 19, 29]. The third group has prominent formation of sarcoid-like granulomas, defined as a nodular collection of epithelioid macrophages with multinucleated giant cells and lymphocytic cuffing associated with large aggregates of corrosion products particles and a mixed macrophagic/lymphocytic infiltrate, possibly representing a subset of patients with distinctive macrophagic features [20, 29]. The fourth group shows perivascular/interstitial lymphocytic infiltrate without macrophagic component [21]. Patients in each implant class were classified based on the predominant histological pattern seen at light microscopy. The rate of appearance of each pattern was compared between the different implant classes.

**Table 2:** Histological patterns analyzed in hip replacement failures due to ALTR

Histological Pattern	Characteristics
Macrophagic Pattern	Macrophagic infiltrate (grade ≥ 1) without or with minimal evidence of interstitial and/or perivascular lymphocytic infiltrate (<grade 1)
Mixed Macrophagic and Lymphocytic Pattern w/wo Plasmacytic Component	Macrophagic (grade ≥ 1) and lymphocytic (grade ≥ 1) infiltrate
Without Presence of Germinal Centers or Eosinophils	
With Presence of Germinal Centers or Eosinophils	
Granulomatous Pattern	Any pattern with predominant presence of sarcoid-like granulomas
Lymphocytic Pattern	Interstitial and/or perivascular lymphocytic infiltrate without evidence of macrophagic infiltrate

### Statistics

All demographic and histological variables were compared across the three implant classes. Descriptive statistics are presented as medians and ranges for continuous variables and as frequencies and percentages for categorical variables. Continuous variables were assessed using the Kruskall-Wallis test. Histological patterns amongst the different implant classes were compared using the Fischer’s exact test. A multinomial logistic regression was performed in order to identify possible predictive factors for the development of the scale of ALTR severity as described in the Campbell’s score. Bonferroni correction was used for pairwise comparisons of histological data adjusted for multiple comparisons.

## Results

### Demographic results

Implant designs that resulted in cases of ALTR in this study are shown in Table 3. Patients in the HRA group were younger in age at time of revision relative to the other implant classes (Table 4). Total implantation time was shortest in the Non-MoM DMNTHA group [median 28 months (range 6–65)], and these patients had a significantly shorter duration of implantation relative to the MoM HRA group [median 48 months (range 5–120); *p* < 0.001] and the MoM LHTHA groups [median 60 months (range 23–132); *p* < 0.001] (Table 4). Duration of symptoms prior to revision did not differ between the different implant classes (Table 4). Preoperative serum cobalt and chromium ion levels were increased in the MoM HRA and MoM LHTHA groups relative to the Non-MoM DMNTHA group (Table 4). Head sizes were larger in the MoM bearing surface groups (HRA and LHTHA) relative to the Non-MoM DMNTHA group.

**Table 3:** Retrieved implants with failure due to ALTR

Non-MoM DMNTHA	Number of Hips
Rejuvenate (Stryker, Kalamazoo, MI)	111
ABG II (Stryker, Kalamazoo, MI)	5
SMF (Smith and Nephew, London, UK)	3
Redapt (Smith and Nephew, London, UK)	2
OTI/Encore R-120 (DJO Surgical, Austin, TX)	1
Aesculap Hip Replacement (Aesculap, Hazelwood, MO)	1
MoM HRA	
Birmingham Hip Resurfacing (Smith and Nephew, London, UK)	36
Cormet Hip Resurfacing (Corin Group, Cirencester, UK)	5
Conserve Hip Resurfacing (Wright Medical Technology, Arlington, TN)	2
ASR Hip Resurfacing (Depuy/Synthes, Warsaw, IN)	1
MoM LHTHA	
Birmingham Hip Replacement (Smith and Nephew, London, UK)	44
ASR Hip Replacement (Depuy/Synthes, Warsaw, IN)	22
Pinnacle Ultramet (Depuy/Synthes, Warsaw, IN)	19
Durom/Metasul (Zimmer, Warsaw, IN)	10
M2a Magnum (Biomet, Warsaw, IN)	8
Profemur (Wright Medical Technology, Arlington, TX)	6
Metal on Metal Bearing S-ROM (Depuy/Synthes, Warsaw, IN)	4
Conserve Hip Replacement (Wright Medical Technology, Arlington, TN)	4
Cormet Hip Replacement (Corin Group, Cirencester, UK)	1

**Table 4:** Demographic characteristics from all three major implant classes

	Non-MoM DMNTHA (*N* = 120 patients)	MoM HRA (*N* = 44 patients)	MoM LHTHA (*N* = 113 patients)
Age (years)	66 (47–87)*	56 (43–75)*	60 (31–84)*
Sex (% female)	64 %	58 %	54 %
Body Mass Index	28 (17–43)^	25 (18–36)^	26 (19–59)
Implantation Time (months)	28 (6–65)°	48 (5–120)°	60 (23–132)°
Symptom Duration (months)	9 (0–60)	12 (0–63)	18 (0–60)
Serum Cobalt	7 (0–169)^#^	16 (1–115)^#^	13 (4–16)^#^
Serum Chromium	1 (0–64)^§^	14 (1–160)^§^	60 (23–132)^§^
Head Size	28 (22–52)^¶^	46 (38–52)^¶^	46 (36–64)^¶^
Cup Size	52 (38–64)	52 (46–58)	52 (48–64)

All values are given as median (range)

*p < 0.005 for the MoM HRA group compared to the other two groups

^*p* < 0.05 for the MoM HRA group compared to the non-MoM DMNTHA group (*p* = 0.005)

°*p* < 0.05 for the non-MoM-DMN THA group compared to the MoM HRA group (*p* < 0.001) and the MoM LHTHA group (*p* < 0.001)

^#^ *p* < 0.05 for the non-MoM DMNTHA group compared to the MoM HRA group (*p* = 0.042)

^§^ *p* < 0.05 for the MoM HRA and the MoM LHTHA groups compared to the non-MoM DMNTHA group (*p* < 0.001) and the MoM HRA group compared to the MoM LHTHA group (*p* = 0.026)

^¶^ *p* < 0.05 for the MoM HRA (*p* < 0.001) and the MoM LHTHA (*p* < 0.001) groups compared to the non-MoM DMNTHA group

### Histological patterns

A summary of the histological patterns seen in each of the three implant classes is shown in Table 5.

**Table 5:** Distribution of the histological patterns in the three implant classes

Implant class	Macrophagic pattern	Mixed Pattern w/o hypersensitivity features	Mixed Pattern w/Hypersensitivity Features	Granulomatous pattern	Lymphocytic pattern
Non-MoM DMNTHA	6	46	32	16	0
MoM HRA	41	48	11	0	0
MoM LHTHA	11	62	22	5	0

All values are expressed as a percentage of total cases of each histological pattern for a specific implant class

**Fig. 2:**
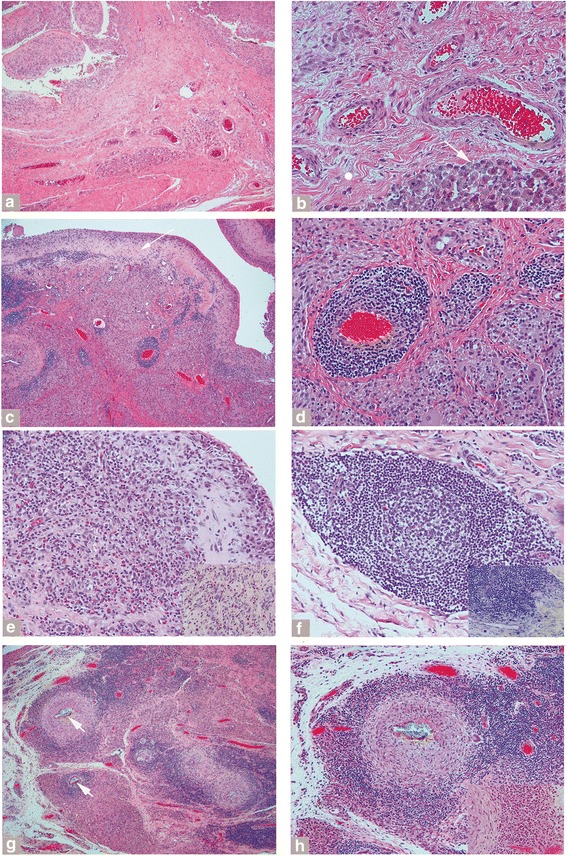
Histological patterns of ALTR. *Macrophagic pattern*: **a** Papillary neo-synovium with macrophagic infiltrate and underlying vascular layer (H-E x50). **b** Vascular layer without lymphocytic infiltrate and cluster of particle laden macrophages, white arrow (H-E x400). *Mixed macrophagic and lymphocytic pattern*: **c** Neo-synovium with superficial macrophagic layer and desmoplastic band, white arrow, and perivascular lyphocytic infiltrate (H-E x50). **d** Perivascular lymphocytic infiltrate associated with large clusters of particle laden macrophages (H-E x200). *Subset of the mixed pattern* *with heightened immunological features*: **e** Neo-synovium with florid interstitial lymphocytic and eosinophilic infiltrate (H-E x200) and association of eosinophils with particle laden macrophages (inset, H-E x400). **f** Perivascular lymphocytic infiltrate with germinal center (H-E x200) associated with numerous mast cells (inset, Toluidine Blue x400). *Granulomatous pattern*: **g** Multiple sarcoid-like granulomas with central aggregate of corrosion products, white arrow (H-E x100). **h** Granuloma at higher power with central collection of epithelioid macrophages and occasional giant cells (H-E x200) and plasmacytic component with binucleated forms admixed with particle laden macrophages (inset, H-E x400)

The macrophagic pattern is characterized by flat to papillary hypertrophic neo-synovium with a variable amount of macrophagic infiltrate and exfoliation of necrotic forms with absence or presence of giant cells containing fine globular and/or irregular aggregates of greenish corrosion products of variable dimension with or without particles of needle-shaped and/or irregular conventional black metallic debris and absent or minimal interstitial and/or perivascular lymphocytic infiltrate (Fig. 2a and b). A thick layer of necrosis is usually not present, although infarction of the neo-synovial papillae or a thin layer of superficial necrosis/infarction can be focally present along with variable foci of foamy macrophages. This pattern was seen in 41 % of cases of MoM HRA failures, but was less common in the other two implant classes (MoM LHTHA, 11 % of cases; Non-MoM DMNTHA, 6 % of cases) (Table 5).

The mixed macrophagic and lymphocytic pattern is characterized by a superficial layer of macrophages with or without an interstitial lymphocytic component, a layer of tissue necrosis/infarction of variable thickness or a band of desmoplastic fibrosis, a variable deep perivascular lymphocytic infiltrate, and macrophages containing fine globular and/or irregular aggregates of greenish corrosion products with or without particles of needle-shaped and/or irregular conventional black metallic debris (Fig. 2c and d). A subset of the mixed macrophagic and lymphocytic pattern shows features usually associated with hypersensitivity/allergy reactions, such as focal or diffuse eosinophilic infiltrate and presence of a large number of mast cells in association with particle-laden macrophages and/or perivascular lymphocytic infiltrate with formation of germinal centers (Fig. 2e and f). Implants with non-MoM bearing surfaces had increased mixed pattern with hypersensitivity features as a percent of total failures (Non-MoM DMNTHA 32 %) versus implant classes with a MoM bearing surface (HRA 11 % of cases and LHTHA 22 % of cases) (Table 5).

The granulomatous pattern is characterized by predominant isolated or confluent granulomas composed of centrally located large aggregates of particulate corrosion products lined or contained by multinucleated giant cells surrounded by a nodular infiltrate of epithelioid macrophages lined by lymphocytic cuff of variable thickness with or without presence of a plasmacytic component (Fig. 2g and h). A granulomatous pattern was most commonly seen in the Non-MoM DMNTHA (16 % of cases) versus the other two implant classes (Table 5).

A significant association (*p* < 0.001) was found with length of implantation and histological classification on univariate analysis, with longer durations of implantation associated with a macrophagic pattern of failure and shorter durations of implantation associated with granulomatous or a mixed pattern with eosinophils and/or germinal centers. Duration of patient symptoms was not associated with histological classification in univariate analysis (*p* = 0.16).

### Morphology results

A summary of morphologic findings among the three classes of implants and the control group is shown in Table 6.

**Table 6:** Significant differences in histological findings from all three implant classes and the control group

	Non-MoM DMN**THA** (*N* = 123 hips)	MoM HRA (*N* = 44 hips)	MoM LHTHA (*N* = 118 hips)	MoP OLTHA (*N* = 31 hips)
Synovial Structure				
Synovial Layer Loss (%)	99	89	99	16.1
Soft Tissue Necrosis (%)	53‡	11‡	32‡	0‡
Sarcoid-like Granulomas (%)	16*	0*	5*	0
Campbell Score (median)	8^§^	5^§^	6^§^	-
Cellularity				
Macrophages (% Grade 1, Grade 2, Grade 3)	7, 51, 42^	0, 5, 95^	1, 34, 65^	3, 16, 81^
Lymphocytes (% Grade 1, Grade 2, Grade 3, Grade 4)	7, 29, 34, 24°	25, 18, 11, 7 °	25, 31, 23, 10°	10, 0, 0, 0°
Plasma Cells (% Grade 1, Grade 2)	26, 18	11, 9	29, 18	0, 0
Eosinophils (%)	20	9	17	0
Macrophage Content				
Polyethylene Particles (%)	2	0	0	80
Metallic Particles (%)	8^#^	39^#^	30^#^	65^#^
Corrosion Products (%)	95	100	99	0
Large Aggregates	74¶	11¶	63¶	0¶
Bone and Bone Marrow	*N* = 58	*N* = 44	*N* = 36	*N* = 0
Necrosis (%)	47	11	23	-
Macrophage Infiltration (%)	57	67	73	-
Germinal Centers (%)	12	2	3	-
Osteolysis (# cases)	4	9	4	-

All values for synovial structure, macrophage content, eosinophils, cell distributions, and bone and bone marrow content expressed as percentage of cases with each morphologic feature

*p = 0.013 for Non-MoM DMNTHA versus MoM LHTHA and MoM HRA

^*p* = 0.007 for OL versus Non-MoM DMNTHA; *p* = 0.007 for MoM HRA versus MoM LHTHA; MoM HRA versus Non-MoM DMNTHA, and Non-MoM DMNTHA versus MoM LHTHA

°*p* = 0.007 for significant difference in distributions of lymphocyte grade between all implant classes except MoM LHTHA versus MoM HRA (*p* = 0.0023)

^#^ *p* = 0.007 for OL versus MoM LTHA and Non-MoM DMNTHA; *p* = 0.007 for Non-MoM DMNTHA versus MoM HRA and MoM LHTHA

^§^ *p* < 0.001 for significant difference in Campbell score between ALTR implant classes

‡*p* = 0.007 for OL versus Non-MoM DMNTHA, MoM LHTHA; *p* = 0.007 for MoM HRA versus Non-MoM DMNTHA; *p* = 0.009 MoM HRA versus MoM LHTHA; *p* = 0.0048 MoM LHTHA versus Non-MoM DMNTHA

¶*p* = 0.011 for OL versus MoM HRA; *p* = 0.007 OL versus Non-MoM DMNTHA and MoM LHTHA; *p* = 0.007 for MoM HRA versus MoM LHTHA and Non-MoM DMNTHA; *p* = 0.026 MoM LHTHA versus Non-MoM DMNTHA

Macrophage distributions were significantly different between the three implant classes, and the MoM HRA group had the highest percentage of cases of grade 3 macrophage distribution (95 % of cases) versus MoM LHTHA (65 % of cases; *p* = 0.007) and the Non-MoM DMNTHA (42 % of cases; *p* = 0.007) (Table 6). Compared with the MoP OLTHA group, the MoM HRA and MoM LHTHA had similar macrophage distributions (*p* = 0.14) (Table 6). Non-MoM DMNTHA had decreased macrophage distributions relative to the MoP OLTHA group (*p* = 0.007) (Table 6). Soft tissue necrosis was more common in the Non-MoM DMNTHA (53 % of cases) relative to the other implant classes (32 % in the MoM LHTHA [*p* = 0.0048], 11 % in the MoM HRA group; *p* = 0.007) (Table 6).

**Fig. 3:**
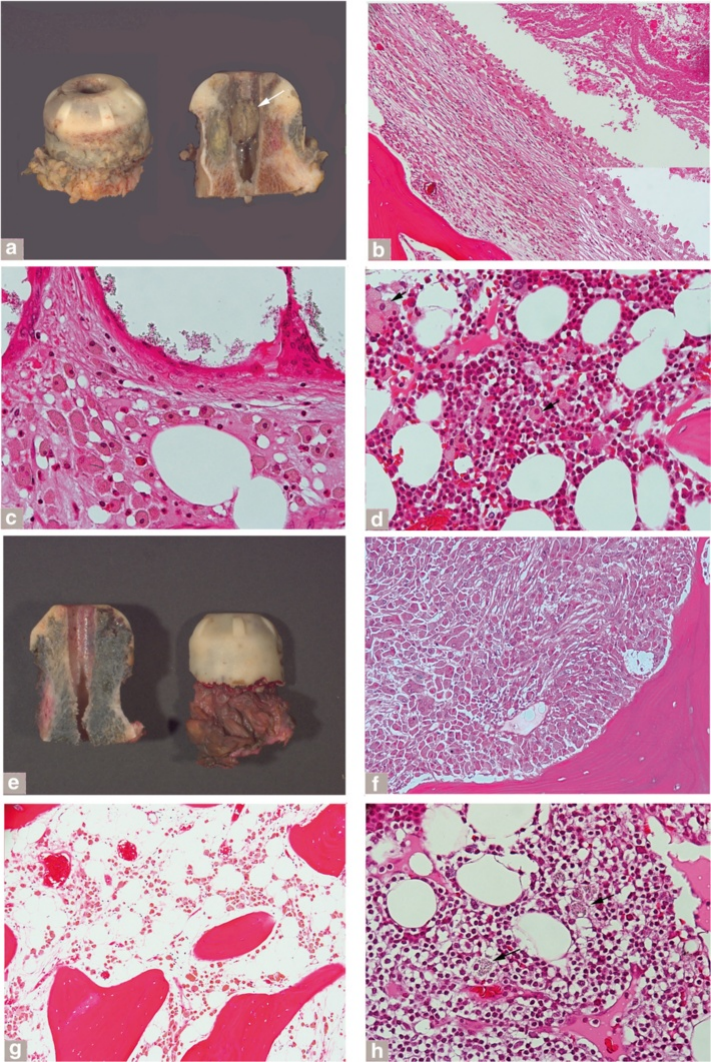
Osteolysis features in the MoM RHA group. **a** Femoral head with orthopedic cement cap lined by papillary neo-synovium and showing osteolytic cavity involving the central groove of the metallic stem, white arrow (Smith and Nephew Birmingham, implantation time 48 months). **b** Content of the osteolytic cavity composed of particle laden macrophages with central exfoliation of necrotic forms in the right upper corner (H-E x100) and detail of the cavity lining cell layer containing greenish corrosion products in inset (x400). **c** Particle laden macrophagic infiltrate under the orthopedic cement cap lined by multinucleated giant cells (H-E x400). **d** Hematopoietic marrow with particle laden macrophages without evidence of lymphocytic reaction (H-E x400). **e** Femoral head with orthopedic cement cap lined by papillary neo-synovium with charcoal grey bone marrow, secondary to diffuse permeation by macrophagic infiltrate with metallic wear content (Smith and Nephew Birmingham, implantation time 78 months). **f** Massive macrophagic infiltrate in bone marrow containing greenish particles of corrosion products and black particles of abrasion metallic wear without evidence of osteoclastic activity (H-E x200). **g** Seeding of particle-laden macrophages in fatty marrow indicative of increased motility (H-E x100). **h** Large aggregate of lymphocytes positive for T-cell (CD3) and B-cell (CD20) marker (not shown) with interspersed particle laden macrophages, black arrows (H-E x 400)

Focal or diffuse macrophagic involvement of the bone marrow was observed in the MoM HRA, MoM LHTHA, and the Non-MoM DMNTHA implants (Table 6). More cases of osteolysis/massive particle laden macrophagic infiltration within retrieved periprosthetic bone samples were seen in the MoM HRA relative to the other implant classes (Table 6). Examination of some of the femoral heads retrieved from failed hip resurfacing implants showed florid particle-laden macrophagic infiltrate in the neo-synovium and massive infiltration of the bone marrow with formation of macroscopically evident, macrophage-lined pseudocystic cavities (Fig. 3a, arrow) with marked exfoliation of necrotic forms (Fig. 3b). Macrophages containing greenish particles of corrosion products (Fig. 3b, inset and 3c) sometimes in association with black particles of conventional metallic debris (Fig. 3e and 3f) were observed streaming from the adjacent neo-synovium and infiltrating the bone marrow forming massive aggregates (Fig. 3f) or small clusters and single forms in the fatty marrow (Fig. 3g) or pushing underneath the orthopedic cement border lined by giant cells (Fig. 3c). In some cases, these were associated with large lymphocytic aggregates (Fig. 3h) or without lymphocytic reaction (Fig. 3d). The presence or absence of lymphocytic infiltrate in the bone marrow usually corresponded to the response seen in the neo-synovial membrane.

**Fig. 4:**
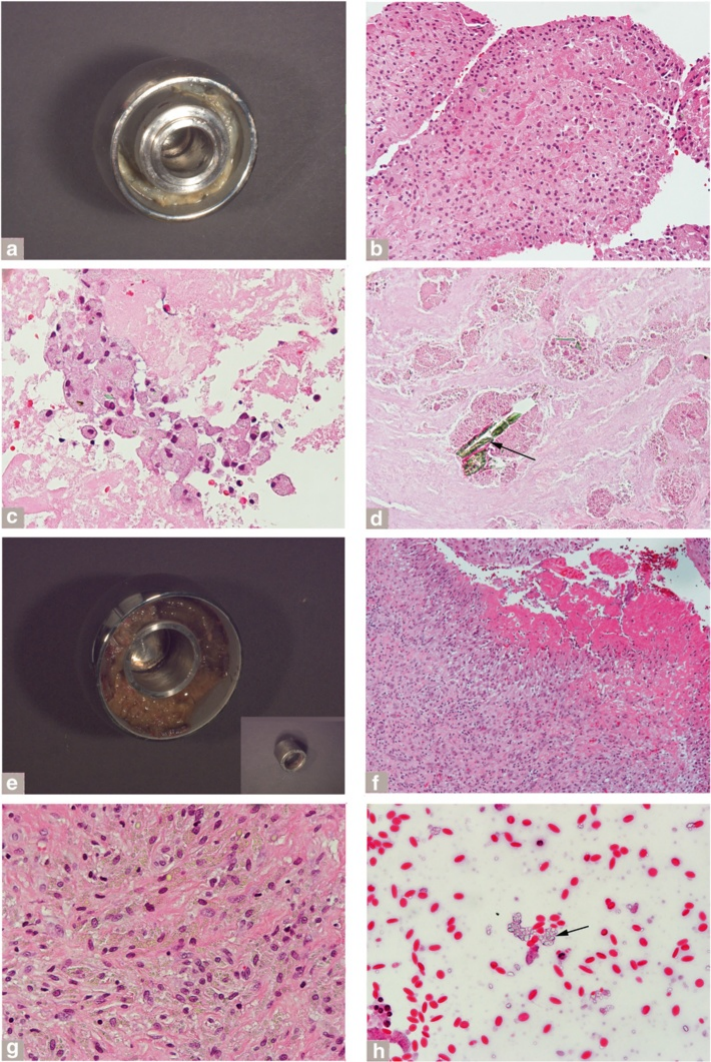
Features of macrophagic pattern in the MoM LHTHA group. **a** Metallic femoral head and inserted metallic adapter sleeve (MAS) with groove filled with dense necrotic cellular debris (DePuy ASR, implantation time 61 months). **b** Papillary neo-synovium with florid macrophagic infiltrate and superficial exfoliation of necrotic forms (H-E x200). **c** Mixture of viable and necrotic particle laden macrophages of necrotic cellular debris shown in (A). **d** Necrotic cellular debris with entrapped large aggregates of greenish particulate corrosion products, black arrow (H-E x400). **e** Metallic femoral head and separate MAS (inset) with groove filled with dense necrotic cellular debris (Smith and Nephew Birmingham, implantation time 44 months). **f** Neo-synovium with florid macrophagic infiltrate and marked exfoliation of necrotic cellular debris (H-E x100). **g** Detail of the macrophgic infiltrate containing globular and irregular aggregates of greenish particulate corrosion products (H-E x400). **h** Cluster of aggregates of pale green corrosion products particles detected in smeared synovial fluid pellet spun at 3,000 rpm x 15 min (H-E x 400)

In the MoM LHTHA, a significant amount of necrotic debris containing macrophagic forms was observed in the metallic femoral head groove of a range of implant manufacturers (Fig. 4a and 4e), deposited from exfoliation of macrophagic viable/necrotic forms containing particles of corrosion products with or without conventional metallic debris (Fig. 4g) from thickened neo-synovial membrane with or without papillary features (Fig. 4b and 4f). Free aggregates of irregular green particles of corrosion products were found in synovial fluid (Fig. 4h, arrow) and in larger aggregates entrapped in necrotic cellular debris (Fig. 4d, arrow).

Lymphocyte distributions were significantly different between the three implant classes, and the MoM HRA group had the lowest percentage of cases of grade 3 and 4 lymphocyte distributions (18 % of cases) relative to the Non-MoM DMNTHA (58 % of cases; *p* = 0.0007), the MoM LHTHA (33 % of cases; *p* = 0.0027) (Table 6). All three ALTR groups had increased lymphocyte distributions relative to the MoP OLTHA group (*p* = 0.007; Table 6). Eosinophils were least common in the MoM HRA (9 % of cases) relative to the Non-MoM DMNTHA (20 % of cases) and MoM LHTHA (17 % of cases), however without reaching significance (*p* = 0.26) (Table 6).

**Fig. 5:**
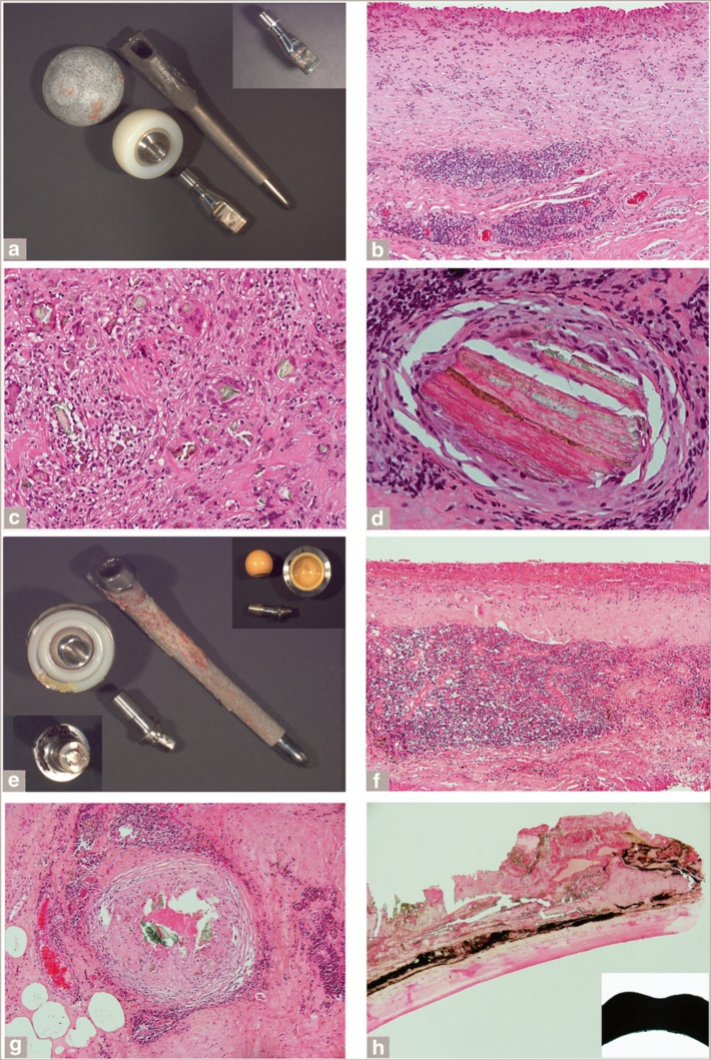
Features of corrosion products in the Non-MoM DMNTHA group. **a** MoP THA with CoCr dual exchangeable neck (inset) with corrosion on distal male taper (Stryker Rejuvenate, implantation time 20 months). **b** Neo-synovium showing superficial layer of macrophagic infiltrate and deep lymphocytic infiltrate (H-E x100). **c** Giant cell reaction without formation of granulomas to large aggregates of greenish and corrosion products (H-E x200). **d** Large aggregate of corrosion products with plate-like structure suggestive of layering of corrosion (greenish) and blood (reddish) products (H-E x400). **e** MoP THA with CoCr dual exchangeable neck with original CoC bearing surface and neck (inset, right upper corner) and second revision neck (inset, lower corner) with corrosion products on the male geared surface (OTI Encore, implantation time at first revision 36 months and at second revision 44 months from first revision). **f** Neo-synovium of first revision showing superficial layer of macrophagic infiltrate and dense layer of lymphocytic infiltrate (H-E x100). **g** Sarcoid-like granulomatous reaction with central green aggregates of corrosion products of second revision (H-E x100). **h** Large aggregate of layered corrosion products detached from the dual exchangeable neck (H-E x100) with scalloped shape of the gearing surface (inset)

The observation of particles of conventional metallic debris was less common in the Non-MoM DMN THA (8 % of cases) relative to the MoM HRA (39 % of cases; *p* < 0.0001), the MoM LHTHA (30 % of cases) (*p* < 0.0001). Corrosion products were seen in either intracellular and/or extracellular locations in almost all cases in each group (Table 6). Extracellular aggregates of these corrosion products were less common in the MoM HRA group (11 % of cases) relative to the Non-MoM DMNTHA (74 % of cases; *p* = 0.007) and the MoM LHTHA (63 % of cases; *p* = 0.007) (Table 6). Examples of large aggregates are shown in two Non-MoM DMNTHAs (Fig. 5a and 5e) with similar histological appearance in the neo-synovial membrane (Fig. 5b and 5f). The large aggregates of particles of corrosion products with plate-like, stratified configuration were present on implant components (Fig. 5a, inset and 5e, lower inset), detachable from the surface (Fig. 5h), or embedded in periprosthetic tissue with breakdown in multinucleated giant cells (Fig. 5c and 5d) with or without a granulomatous histological pattern irrespective of implant bearing surface (Fig. 5g and 5d). Sarcoid-like granulomas were more likely to be present in the Non-MoM DMNTHA (16 % of cases) than in the MoM LHTHA (5 % of cases; *p* = 0.013) and MoM HRA (0 % of cases; *p* = 0.013) (Table 6).

Median Campbell (ALVAL) score was lower in implants with MoM bearing surfaces (MoM HRA, median score 5 and LHTHA, median score 6 relative to the Non-MoM DMNTHA, median score 8 (*p* < 0.001). A multinomial logistic regression was performed in order to examine the association between preoperative demographic variables (age, sex, BMI, implant type, duration of symptoms, duration of implantation) with Campbell’s ALVAL score at revision. After adjustment, age (*p* = 0.010) and implant type (*p* = 0.002) were the only variables independently associated with Campbell’s ALVAL score at revision. The MoM HRA group was an independent factor for a lower score at revision, using MoM bearing surfaces as a reference.

## Discussion

The occurrence of ALTR has been described in cases series for all three classes of implants analyzed in our study [4, 5, 13–15, 24, 27, 30, 32–46]. Recent reports have shown that the histological patterns of ALTR are more diverse than the original description of ALVAL and this complexity may result in different mechanisms of failure, which can have clinical implications for patient surveillance and outcomes after revision arthroplasty [13, 17, 20, 21, 29, 46]. The purposes of this study were to describe the frequency of different histopathological patterns of soft tissue failure in ALTR, their association with different implant class, and the association of histopathological findings with clinical features of implant failure.

Our histological analysis demonstrates that ALTR encompasses a range of histological patterns ranging from purely macrophagic to mixed lymphocytic and macrophagic with or without features of associated with hypersensitivity (eosinophils/mast cells and/or lymphocytic germinal centers), and predominant sarcoid-like granulomas as previously described [13, 17, 19–21, 29, 46]. This is the largest study to the best of our knowledge to classify the histological patterns of ALTR across a diverse range of implants and its association to their clinical performance.

### Macrophagic pattern

**Fig. 6:**
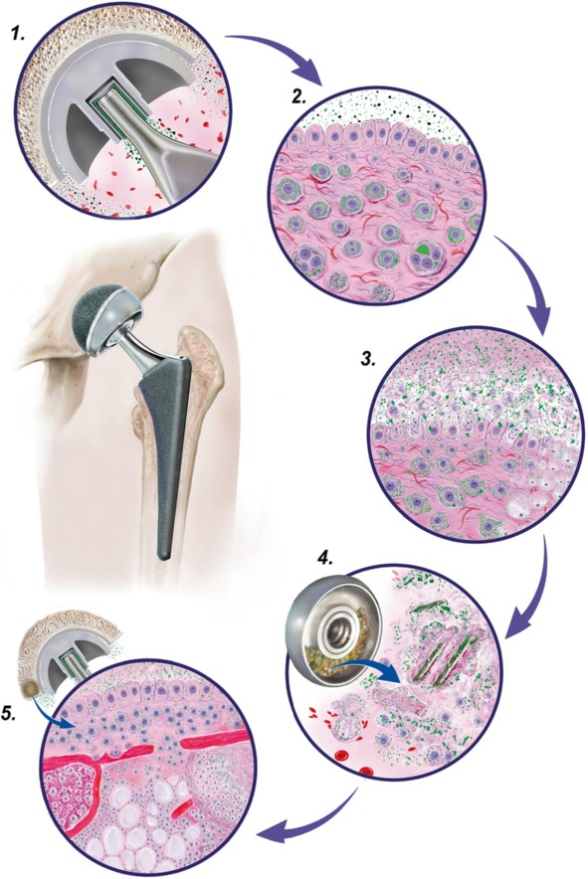
Phases of macrophagic pattern of ALTR in MoM LHTHA prosthesis. *Circle 1:* Nano-scale particles generated at the bearing surface by sliding tribocorrosion and nano and micron-scale at the metallic adapter sleeve-femoral neck surface by fretting/crevice corrosion where larger aggregates are formed. Conventional abrasion metallic wear can also be generated at the bearing surface by edge loading and/or neck junction at any time of implantation. *Circle 2:* Phagocytosis/pinocytosis of particulate material by neo-synovial superficial and deep layer macrophages with multinucleated giant cells containing large particulate aggregates. *Circle 3:* Massive apoptosis of neo-synovial particle-laden macrophages through oxidative stress with formation of degenerated foamy forms (right side) and release of necrotic cellular debris and secondary particles of corrosion products/abrasion metallic particles with disruption of cytoplasmic phagosomes. *Circle 4:* Accumulation of viable macrophages, necrotic cellular debris, red blood cells, and entrapped small and large aggregates of primary and secondary particles of corrosion products in the femoral head groove with substantial increase in thickness of the synovial fluid and subsequent modification of its lubrication properties. *Circle 5:* Bone marrow involvement by neo-synovial particle-laden macrophagic infiltrate through resorptive osteoblastic activity and direct invasion through cortical gaps with formation of osteolytic cavity (right side), diffuse seeding of the fatty marrow (middle area), and involvement of the hematopoietic marrow (left side)

Our results confirm that a macrophage predominant pattern of soft tissue failure exists in ALTR as previously reported, and it occurs more commonly in implants with MoM bearing surfaces [20, 21, 29]. We hypothesize that this is related to surface corrosion generating nanoparticle size wear debris unique to this bearing surface, as originally observed and later characterized by transmission and scanning microscopy [15, 18]. Phagocytosis/pinocytosis of metallic nanoparticle debris into cytoplasmic phagosomes with subsequent release of metallic ions has been shown to produce high level of oxidative stress in macrophages, resulting in a marked increase in reactive oxygen species promoting protein carbonylation, a well-known consequence of cellular oxidative stress, leading to a loss of biological function and ultimately cell death [47]. This process may be accelerated and enhanced by the addition of corrosion wear particles generated at the head-neck taper surface through the interposition of a CoCr metallic adapter sleeve. A proposed mechanism of implant failure of ALTR with the macrophagic pattern due to soft tissue and bone involvement exemplified for a MoM LHTHA is illustrated in Fig. 6. We hypothesize that massive corrosion product-associated macrophage apoptosis, exfoliation of necrotic cellular debris and phagocytized secondary wear particles into the joint fluid alters the bearing surface lubrication in implants with a MoM bearing surface. This failure mechanism would be difficult to replicate in any in-vitro tribology system or constructed tribocorrosion test apparatus [48, 49]. These alterations of the tribological film may lead to accelerated corrosion of the bearing surface and formation of abrasion-induced metallic wear. Clinically, this process may manifest as patient-reported mechanical symptoms that develop years after implantation along with increased serum metallic ion levels as the bearing surface is no longer properly lubricated. Previous studies have also shown that elevated metal ion levels occur due to implant misalignment; however, implant positioning does not account for many clinical failures, and this mechanism could provide an alternative explanation [50].

The occurrence of macrophagic bone marrow infiltrate with or without associated histological evidence of osteolysis in the MoM HRA class may be explained by three different mechanisms: 1. The well- studied osteoclastic activation; 2. Increased macrophagic motility with mass burden necrosis and formation of pseudocystic cavities in the acetabular and/or femoral bones; 3. Penetration of corrosion particles and viable macrophages pushed by lubrication fluid pressure during motion. This component of the ALTR has been overlooked, but could become clinically significant with extended time of implantation and corrosion wear particle generation, especially for MoM HRA and MoM LHTHA groups [51, 52].

### Mixed macrophagic/lymphocytic pattern

Similar to previous studies, we found a mixed lymphocytic and macrophagic pattern to be common in ALTR however, within this group, the range of cellular infiltrates and tissue morphology suggests that individual variation exists within this pattern. Specifically, we have found the presence of mast cells/eosinophils and/or formation of lymphocytic germinal centers usually associated with tall endothelial cell venules in a subset of patients within this group. Mast cells are difficult to be identified in a crowded inflammatory background with conventional histology, although their presence has been previously demonstrated by immunohistochemistry [17]. The increased presence of mast cells, eosinophilic infiltrate, and lymphocytic germinal centers may be an expression of hypersensitivity/allergy to particulate conventional metallic or corrosion debris in certain subsets of patients. Previous authors have noted a weak correlation between wear characteristics and soft tissue response in a subgroup of patients with ALTR [29, 39]. Subsets of patients with evidence of neo-synovial tertiary lymphoid organs or sarcoid-like granulomas have been noted by previous authors, and these all may represent patient-specific variable immune responses to particulate corrosion debris [17, 19, 20]. Identification of patients with hypersensitivity to metal debris in joint replacement remains controversial because skin patch testing and lymphocyte transformation testing does not reliably predict patient-specific implant performance [53–55]. Systemic toxicity such as cardiomyopathy, neuropathy, and dermatological manifestations has been reported in limited case series, and these findings are typically associated with very high serum ion levels, particularly cobalt [56]. Recent work has shown a prominent up-regulation of interferon gamma associated chemokine expression in ALTR with a mixed lymphocytic and macrophagic pattern [57]. Activation of hypoxia-inducible factor secondary to cellular oxidative stress has also been implicated in this process [47, 58, 59]. Further studies on the molecular signaling pathways involved in ALTR are critical.

Similar to other non-specific foreign body responses, a pure lymphocytic pattern was not observed in our study, and macrophagic phagocytosis of wear particles is a key initial event. This activation of the innate immune system may or may not be associated with subsequent involvement of an adaptive immune response, which may in turn lead to further macrophagic recruitment [29]. We believe that the absence of particle laden macrophages in some reported cases may be related to tissue sampling rather than true absence of such cells from the affected tissues [21].

### Granulomatous pattern

The granulomatous pattern was observed in both THA groups with variable frequency and not in the MoM HRA group. We hypothesize that it requires the presence of large aggregates of particulate corrosion products, which is seldom present in the MoM HRA group. This pattern represents a distinctive patient-dependent macrophagic response which might be similar to the granulomatous reaction observed in sarcoidosis and triggered by exposure to various microbial agents.

### Use of Campbell’s ALVAL scoring system

Currently, the Campbell’s ALVAL score has been the primary method to assess ALTR in the periprosthetic soft tissue, showing good correlation with MRI studies [28, 60]. Using multinomial logistic regression, we found that implant configuration was associated with the Campbell’s ALVAL score. In particular, hip resurfacing was associated with a lower score at revision for ALTR. In our experience the use of the score has limitations in ALTR because it is focused primarily on necrosis, scored twice in the synovial lining and tissue organization sections with a maximum of 3 points each, and the lymphocytic infiltrate, which is given a maximum of 4 points in a total maximum score of 10 [13]. The predominantly macrophagic pattern of soft tissue failure would produce low Campbell’s ALVAL scores due to no or minimal lymphocytic infiltrate and no necrosis, but can still result in soft tissue arthroplasty failure. There is no grading of the macrophagic exfoliation and no consideration for macrophagic involvement with or without associated osteolysis in the femoral/acetabular bone marrow, which may have significant clinical implications for implant performance.

### Public health implications

Our study suggests that the histological analysis of periprosthetic tissue in cases of ALTR can provide information that may be useful for longitudinal monitoring of implants. For example, we found that mixed lymphocytic and granulomatous subtypes were associated with shorter durations of implantation and were more common in the MoM LHTHA and Non-MoM DMNTHA with a known occurrence of taper corrosion [5, 7–9, 27, 61, 62]. In contrast, the predominantly macrophagic pattern is more common in the MoM HRA group which generates nano-size corrosion/conventional metallic debris particles only at bearing surface.

The association between histological classification and time to revision may have clinical implications because implants with high number of patients with mixed macrophagic/lymphocytic pattern may fail earlier due to formation of pseudotumors with soft tissue necrosis, and this has resulted in implant recalls, such as the Stryker Rejuvenate and ABGII models. Implants with predominant macrophagic pattern, may fail at medium-long implantation time at an undetermined rate due to changes in the tribological lubrication process and/or macrophagic driven osteolysis. This unpredictable risk at the present time would call for a follow-up program with a frequency and modalities to be determined coupled with studies aiming at identifying biological and cellular factors associated with this type of adverse reaction [52, 63].

Our analysis showed that similar patterns of ALTR were present in implant classes of similar configuration and material composition independent of the manufacturer. This suggests the need for prompt observation and monitoring of any class of implants exhibiting a pattern of early failure with immediate reporting of sentinel cases to regulatory agencies/implant registries with the aim of avoiding high rates of complications for a large number of patients. Additionally, our results have made a case for the inclusion of the pathology report of revision cases in hospital based, regional, and national implant registries as an important and valuable tool in assessing modalities of implant failures along with the implementation of an international consensus classification, as the one recently reported for the periprosthetic soft tissue [64].

### Study limitations

We acknowledge several limitations with the current study. The first and most important is that our analysis is based on our hospital osteolysis/adverse local reaction tissue and repository database, which depends on the patient population admitted to the hospital and histological examination at surgical implant revision end-point. Our hospital serves as a tertiary referral center for revision arthroplasty cases; therefore, we cannot determine the overall class or device-specific implant performance from our data. The second is the attempt to reconstruct the natural history of the adverse reaction based on a single observation at the time of implant revision, although partially compensated for by the extensive tissue sampling. The third is the absence of the following sets of clinical data: a. physical activity pre and post-operative, although it has shown a weak correlation to elevated serum metal ion levels, suggesting that activity-related bearing surface wear plays only a minor role in elevated serum cobalt or chromium levels [65, 66]; b. pre and post-operative bone density, which may influence the occurrence/rate of implant mechanical loosening/osteolysis especially in the female population which requires a sophisticated method for proper assessment, such as high-spatial-resolution bone densitometry with dual-energy X-ray absorptiometric region-free analysis [67], which is not currently performed as standard of care at our institution; c. wear analysis by biomechanics examination of the metal-on-metal implants for surface roughness, although retrieval analysis and blood metal measurements contribution to the understanding of ALTR has been previously addressed in a comprehensive review and no clear dose–response relationship between wear and ALTR could be established [68].

## Conclusions

ALTR encompasses a diverse range of histological patterns, which are reflective of both the implant configuration independent of manufacturer and clinical features such as duration of implantation. The predominant macrophagic pattern and its mechanism of implant failure represent an important subgroup of ALTR which could become more prominent with increased length of implantation. Further studies should characterize the physical and chemical characteristics of wear particles and the molecular characteristics of the generation and development of these different histological patterns of ALTR and relevant mechanisms of failure in different implant classes and/or specific devices.

## Abbreviations

THA: Total hip arthroplasty
ALVAL: Aseptic lymphocyte dominated vasculitis associated lesion
ALTR: Adverse local tissue reaction
ARMD: Adverse reaction to metallic debris
MoP: Metal-on-polyethylene
CoP: Ceramic-on-polyethylene
DMN: Dual modular neck
MoM: Metal-on-metal
LHTHA: Large head THA
HRA: Hip resurfacing arthroplasty
